# Is Sensible Heat Flux Useful for the Assessment of Thermal Vulnerability in Seoul (Korea)?

**DOI:** 10.3390/ijerph17030963

**Published:** 2020-02-04

**Authors:** You Jin Kwon, Dong Kun Lee, You Ha Kwon

**Affiliations:** 1 Interdisciplinary Program in Landscape Architecture, Seoul National University, Seoul 08826, Korea; eugeneugene.kwon@gmail.com; 2 Department of Landscape Architecture and Rural System Engineering, Seoul National University, Seoul 08826, Korea; 3 Department of Physical Medicine and Rehabilitation, TS Plastic Surgery Hospital, Rex Tower 12-13F, 108 Dosan-daero, Gangnam, Seoul 06038, Korea; ceo@tsprs.com

**Keywords:** sensible heat flux, thermal comfort and health, sensible heat vulnerability, urban heat island effect, heat-related mortality rate, heat vulnerability index, thermal environment, health

## Abstract

Climate change has led to increases in global temperatures, raising concerns regarding the threat of lethal heat waves and deterioration of the thermal environment. In the present study, we adopted two methods for spatial modelling of the thermal environment based on sensible heat and temperature. A vulnerability map reflecting daytime temperature was derived to plot thermal vulnerability based on sensible heat and climate change exposure factors. The correlation (0.73) between spatial distribution of sensible heat vulnerability and mortality rate was significantly greater than that (0.30) between the spatial distribution of temperature vulnerability and mortality rate. These findings indicate that deriving thermally vulnerable areas based on sensible heat are more objective than thermally vulnerable areas based on existing temperatures. Our findings support the notion that the distribution of sensible heat vulnerability at the community level is useful for evaluating the thermal environment in specific neighbourhoods. Thus, our results may aid in establishing spatial planning standards to improve environmental sustainability in a metropolitan community.

## 1. Introduction

Approximately 48% of the global population will face lethal heat waves by 2100 due to rising global temperatures associated with climate change [1]. Moreover, as urbanization accelerates [2], researchers have raised concerns regarding the deterioration of the thermal environment [3,4]. Several studies have investigated the urban heat island (UHI) effect [5,6,7] in efforts to address global warming due to urbanisation [8]. Additional studies have examined the influence of heat waves on heat-related mortality [9,10].

Ebi et al. argued for the need to improve analytical techniques for exploring and identifying vulnerabilities due to urban warming [11]. The UHI effect represents a serious threat to citizens of population-concentrated metropolitan areas [12,13] where urban canyons exacerbate thermal conditions and increase heat concentration [14,15]. Moreover, recent studies have assessed heat vulnerability mainly in Europe and the United States [16] in regional scale and shown the trend of using various methods; a principal component analysis, a regression, and multi-criteria outranking approach [17,18,19]. Therefore, it is necessary to review thermal vulnerability among residents in urban and metropolitan areas at the community level [20,21]. 

However, Birkmann defined the vulnerability as physical, social, economic, environmental and institutional mechanisms that determine systems’ susceptibility and dealing and adaptive capacity considering how the systems react to dangers, such as the effects of climate changes [22]. Thermal vulnerability has been popularly assessed by heat events based on summer temperature [21,23]. Since temperature is the result of heat exchange, we need to understand the mechanism of space and heat flux to find adaptation methods in thermally vulnerable areas [24]. The aim of this study was to compare whether sensible heat flux, which is more effective than temperature in quantitative thermal environment analysis [25,26], is more effective than temperature in vulnerability assessment.

In the present study, we defined vulnerability based on sensible heat flux as “sensible heat vulnerability” and vulnerability evaluated by temperature as “temperature vulnerability” to distinguish the two different concepts of thermal vulnerability from the general thermal vulnerability used by previous studies. By comparing sensible heat and temperature vulnerabilities in an urban space, it is possible to detect the accurate thermal vulnerability to improve urban thermal vulnerability effectively at the community level. 

Such assessment of urban heat vulnerability contributes toward sustainable development in the community. Communities should have determinants and advanced assessment information on vulnerability to urban warming [11]. Other studies have evaluated the impact of the UHI effect on health [27] and thermal comfort [28,29]. The influence of personal characteristics on health outcomes related to the thermal environment has also been examined in several studies [30,31]. Thus, researchers have evaluated vulnerability [32], vulnerability indices reflecting social statistical data [16], and surface temperature via remote sensing of various heat-related indices [33]. Furthermore, studies have investigated the relationship between heat and aging [34] as well as thermal vulnerability in children [35]. Given that the observed temperature increases in population-concentrated cities, researchers have recently focused on populations exhibiting extreme vulnerability to urban heat (e.g., children and older adults) who are difficult to cope with the heat event promptly [36]. These studies have aimed to examine the impact of urban green space on thermal relaxation in children [37], offering approaches to urban design that mitigate the daytime UHI effect in high-density urban environments by incorporating a green space [38].

One recent study examined strategies for mitigating the UHI effect to improve thermal comfort and health during heat waves [39]. Studies on spatial characteristics of specific neighbourhoods have greatly aided in addressing the issue of thermal vulnerability and reported the influence of spatial characteristics (e.g., urban canyon structure) on the UHI effect [40]. Heat-related mortality due to the influence of climate change has been addressed in multiple countries, communities, and complex environments [41,42,43]. The effects of temperature on mortality and health policies have also been addressed [44], in addition to the spatial causes of vulnerability in areas with persistent heat waves [45,46]. Furthermore, researchers have examined the impact of altering land cover in reducing temperature and radiation [47].

Urban heat islands have an impact on urban warming, and thus, measures are needed to mitigate the casualties caused by summer heat waves in the city. To solve the increasing thermal vulnerability of cities with high population density but not forced movement, solutions for improving spatial thermal comfort have been studied. Therefore, the purpose of this study is to analyse the vulnerability through sensible heat flux, a physical concept of space, and to easily apply physical and quantitative approaches to alleviate heat island effect.

Multiple studies have addressed the impact of urban greenery and land cover on temperatures [48], as well as the spatial distribution of sensible and latent heat flux [49]. Further studies have described the quantitative relationship between physical space and radiant heat, noting that buildings and artificial surfaces in the urban space increase radiation and sensible heat flux [50]. From the previous reviews, physics studies have also reported the relationship between sensible heat flux and temperature [51]. Research has further indicated that the impact of temperature on mortality risk is proportional to the length of thermal episodes [52]. Temperature increases are more dangerous in urban areas than in urban outskirts, whereas hazardous thermal areas may be scattered throughout the city [53]. The degree of urban sprawl also influences the distribution of hazardous thermal areas [54]. However, an integrated socioeconomic response strategy for climate change is critical [55]. To cope with the progress in urbanisation, it is becoming increasingly necessary to elucidate environmental characteristics that influence thermal vulnerability to reduce the impact of the UHI effect and heat waves.

Currently, studies regarding thermal vulnerability in a given area are derived from: (1) a sensitivity based on population, socioeconomic indices, (2) an ability to adapt to high temperatures according to the level of medical infrastructure, and (3) a climate change exposure based on climate-related variables of heat, temperature and spatial attributes. Assessments of thermal vulnerability rely on the three indices; sensitivity, adaptive ability and the exposure, including socioeconomic vulnerability, isolation of older adults, and the number of unrecognised areas, which strongly influence surface temperature [56]. Some research, which includes the three indices, has also suggested that the thermal vulnerability index is related to environmental factors [57], with several researchers attempting to verifying statistical relationships for thermal vulnerability [58,59,60].

One major factor that cannot be overlooked is the response to heat. In other words, heat may differ due to differences in thermal sensitivity based on age and personal characteristics. Such differences complicate the scope and scale of heat waves in urban areas [61], although researchers have developed a web-based tool for combining and mapping the vulnerability index among older adults [62]. Older adults living alone, preschool children, and patients with heat-related illnesses living in a community are considered vulnerable or sensitive to high temperatures or sensible heat flux [63,64]. Sensible heat flux can be mitigated by increases in the number of medical institutions or green spaces in the community. Thus, thermal vulnerability due to external stresses can decrease depending on the level of social efforts. In general, vulnerability can be reduced by mitigating external stresses or by strengthening internal adaptive capacities [65]. The sensitivity of sensible heat flux is important for assessing vulnerability in various populations [66].

In order to identify and mitigate vulnerability, most of all, analyses of extreme urban heat events must take spatial characteristics into account [10]. Spatial mapping of vulnerability can be improved using a meso-scale approach to city-level units [32]. That is, it is necessary to approach vulnerability at the community level [20].

According to previous reviews, we found that the current method for identifying vulnerable areas is based on temperature and therefore does not reflect the heat budget. As urban heat can be influenced by sensible heat flux or heat balance [67], we aimed to compare methods for assessing vulnerability based on existing temperatures after examining whether the current method, which is based on heat budget as determined using spatial characteristics, can be utilised for vulnerability assessments. 

For this reason, sensible heat flux is an important variable for estimating thermal vulnerability. Sensible heat flux, especially that related to heat yields, depends of the type of land cover. Therefore, extracting heat-sensitive areas based on the sensible heat flux makes it easy to identify the ratio of land cover. Diverse methods can be used to secure heat comfort [68,69]. Given the relationship between heat balance and thermal comfort [70], heat flux can aid in a spatial action planning to improve the thermal environment at a community scale. However, there are two important questions concerning this approach. First, can sensible heat flux at the community level be used to conduct vulnerability index that accurately reflect the thermal environment? Second, which variable is easier to identify vulnerable areas, sensible heat flux or temperature? 

In the present study, we aimed to identify a more reasonable method for evaluating thermal vulnerability by comparing areas with high sensible heat flux and areas with high temperature using two methods. One method relied on heat budget to determine sensible heat, whereas another relied on temperature. If the vulnerable areas identified based on heat budget and temperature are similar, our results should support the adoption of a new vulnerability index that incorporates spatial attributes. Thus, we verified thermal vulnerability based on sensible heat flux by comparing spatial distribution patterns (maps) of mortality rates and vulnerability at a community level.

## 2. Materials and Methods

### 2.1. Case Study

Seoul (37.33° N 126.58° E) is a global mega-city with a population density of 27,018 people/km^2^ [71]. It is the capital of Korea and home to approximately 19% of the total population (9,780,000/51.47 million) (2017, Statistics Korea). Approximately 65% (366 km^2^) of the city’s surface area is covered by artificial surfaces. Summers in Seoul are characterised by heavy rain (precipitation: 892.1 mm), whereas winters are relatively dry (precipitation: 67.3 mm). Since heatwaves vary in duration, intensity, or temperature depending on where they occur, there are naturally various definitions of heatwaves [72,73]. Therefore, studies that set the threshold through health indicators have recently been performed. Although the average daytime temperature in the summer is 32 °C, temperatures can reach as high as 37 °C. Summer in Seoul begins in mid-June and lasts until early September. In 2015, the number of heat-wave days in Seoul was 5.9 days in August, which was higher than the average number of annual heat-wave days (5.3 days) [74]. In contrast, the numbers of heat-wave days for June and July were 0.6 and 3.2 days, respectively (Korean Meteorological Administration, KMA). The term “heat-wave” does not have a universally consistent definition but we considered heat-waves as temperatures of 33 °C or higher for 2 or more days; in addition, heat-wave days refer to the number of days with the highest daily temperature above 33 °C according to the KMA [75]. Thus, in this study, the number of thermal mortalities was counted from the second day from the start of the heat wave [18]. The number of patients experiencing heat-related illness during the summer in Seoul continues to increase each year. In urban areas, which are vulnerable to the UHI effect, these effects are further amplified. Notably, the UHI effect also continues to increase in Seoul due to the widespread presence of impervious surfaces and decreases in green space [76]. 

### 2.2. Method for Assessing Thermal Vulnerability

**Figure 1:**
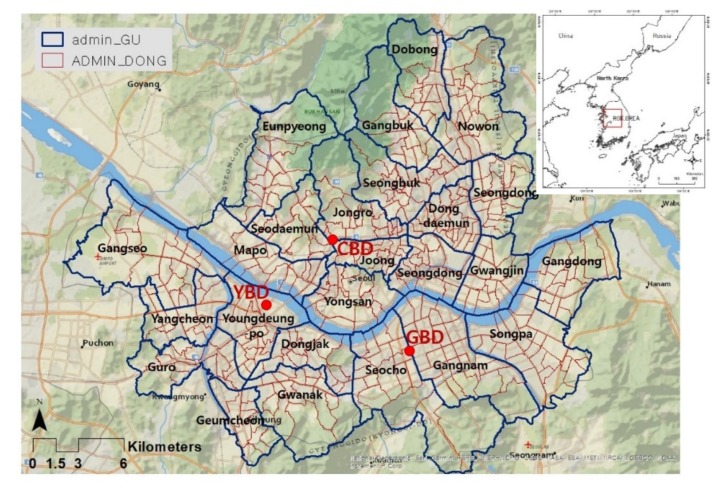
Map of Seoul. Note: CBD refers to a traditional business district. YBD indicates Yeoido business district, and GBD stands for Gangnam business district. These three districts are the main urban centres in Seoul [77]. The variable “admin_GU” represents 25 administrative districts and “ADMIN_DONG” refers to neighbourhood administrative districts. Source: GIS map (https://sgis.kostat.go.kr).

In the present study, we analysed the 2015 Geographic Information System (GIS) spatial data for 438 neighbourhood administrative districts provided by an open Seoul database and the Korean Statistical Information Service (KOSIS); the ratio of five landcovers and administrative boundary (Figure 1, Appendix B). 

**Figure 2:**
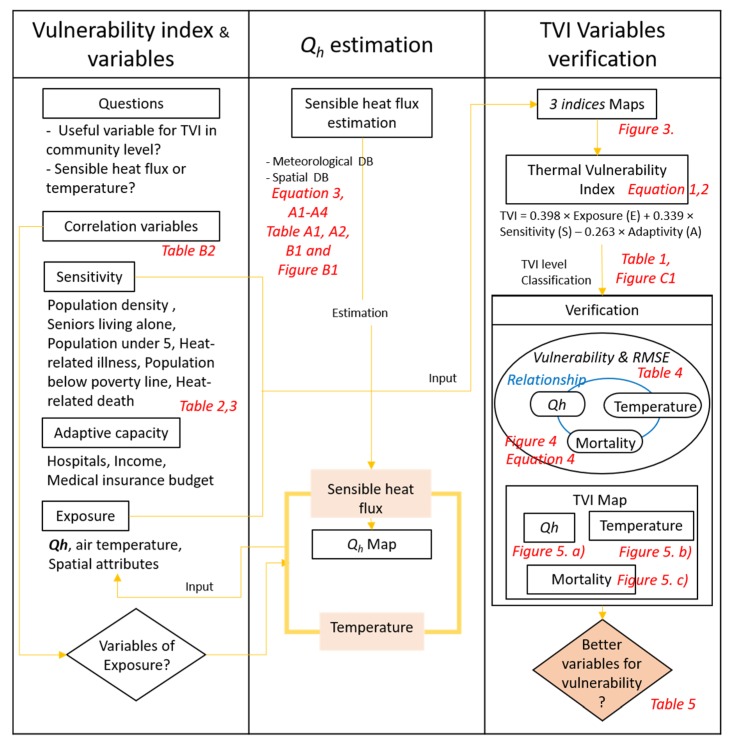
Flow chart of research methods.

To evaluate thermal vulnerability, we performed a correlation analyses of indices used in previous studies [78]. Our study was focused on the suggestion of other variables for investigating thermal vulnerability for thermal environment improvement. There are three parts of the research; vulnerability index and variables, sensible heat flux (Q_h_) estimation and thermal vulnerability index (TVI) variables verification (Figure 2). Firstly, we tested correlation of variables based on previous researches related to the thermal vulnerability (Appendix B Table A4). Then, we conceived variables of the exposure for the thermal vulnerability from comparing temperature-based vulnerability and sensible heat flux-based vulnerability. 

In the second part, we estimated the sensible heat flux in community scale (Equations (3), (A1)–(A4), Table A1 and Table A2) for the comparison in the first part. In this step, we rasterised the sensible heat flux based on existing data (Appendix A and Appendix B), considering the energy balance, net radiation, latent heat, sensible heat, storage heat, and artificial heat [24]. Then, we used the heat flux as a variable of sensible heat vulnerability. 

**Figure 3:**
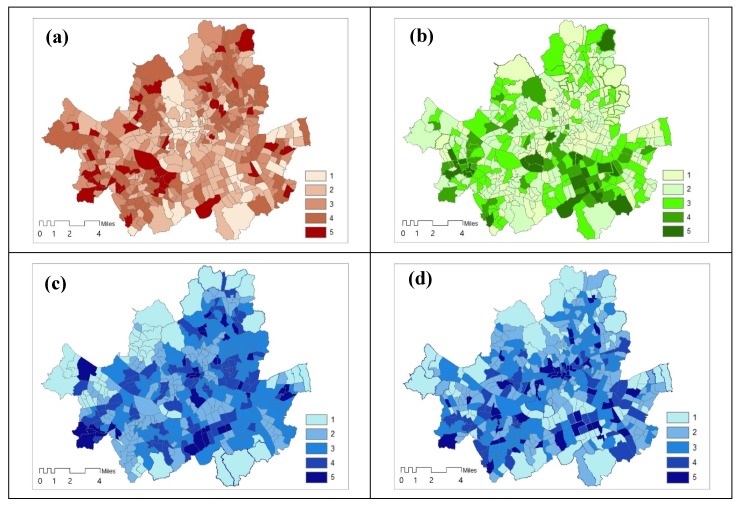
Three indices of temperature and sensible heat flux. **Note:** (**a**) Sensitivity to both temperature and sensible heat; (**b**) Adaptive capacity to both temperature and heat; (**c**) Exposure to temperature; (**d**) Exposure to sensible heat; Standardised value class (range): 1 (0~0.20), 2 (0.21~0.40), 3 (0.41~0.609), 4 (0.61~0.80), 5 (0.81~1.00).

**Figure 4:**
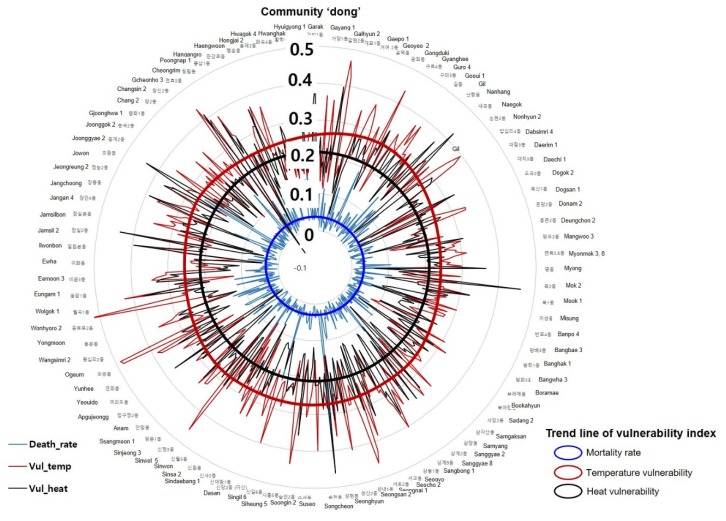
Mortality rate in relation to vulnerability based on temperature and sensible heat flux. Note: Vul_temp (red): temperature vulnerability (vulnerability based on temperature); Vul_heat (black): sensible heat vulnerability (vulnerability based on sensible heat flux).

**Figure 5:**
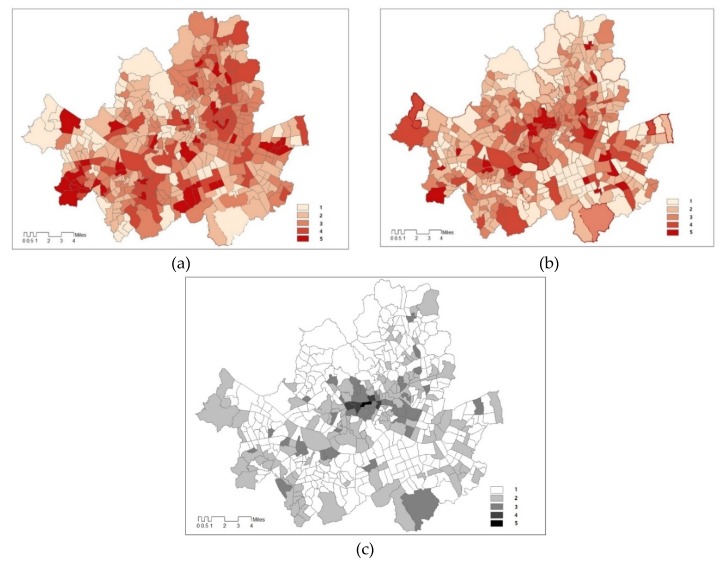
Vulnerability to heat flux and temperature in relation to mortality rate. (**a**) Temperature Vulnerability index; (**b**) Sensible heat vulnerability index; (**c**) Heat-related illness mortality rate. Note: Clustering Class (range): 1 (0~0.08), 2 (0.08~0.32), 3 (0.32~0.49), 4 (0.49~0.76), 5 (0.76~1.00). Because there are none values ( = 0) on mortality rate in communities, the range of the both sensible heat and temperature vulnerability was classified using hierarchical clustering to compare with the death rate.

**Figure A2:**
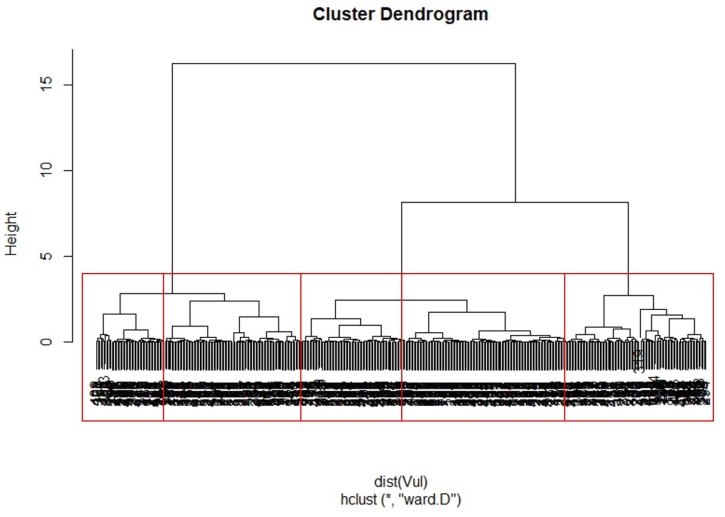
Calinski-Harabasz (C-H) Index Analysis for the level range of vulnerability.

In the third part, we rasterised three indices maps and found a different pattern of the exposure index between sensible heat flux-based map and temperature-based map (Figure 3). Then, we derived thermal vulnerability index (TVI) (Equations (1) and (2)), classified in five levels for the rasterization and comparison (Table 1, Appendix C Figure A2). To verify the TVI variables, we compared a trend line of sensible heat vulnerability and temperature vulnerability with correlation of two variables’ vulnerability (Figure 4, Equation (4)). Lastly, answering to the research question, we performed validation comparing the sensible heat and temperature vulnerability index maps, and mortality rate map in community scale (Figure 5).

### 2.3. Thermal Vulnerability and Thermal Vulnerability Index (TVI)

IPCC defines the vulnerability as “the degree to which a system is susceptible to, or unable to cope with, adverse effects of climate change, including climate variability and extremes.” Thus, heat vulnerability is “a function of the character, magnitude, and rate of heat variation to which a system is exposed, its sensitivity, and its adaptive capacity” [79]. Vulnerability to heat has been estimated based on the combination of climate change exposure (E), sensitivity (S), and adaptive capacity (A) [21,65,80,81,82]. However, in the present study, we aimed to determine whether indices other than temperature can be used to assess thermal vulnerability. Therefore, in contrast to previous studies, we compared the ability of high temperature and high sensible heat flux and spatial attributes as the exposure variables for determining vulnerability. 

In the present study, thermal vulnerability was determined based on external stresses, such as sensible heat flux, temperature [56,83], and the influence of the built environment. External stress is a parameter that indicates the extent to which heat is exposed to space and is among the components used to calculate thermal vulnerability, which reflects human sensitivity and adaptive capacity to heat. Temperature was defined as the average temperature during the day (between 12:00 and 16:00) in a community scale, whereas maximum temperature was defined as the highest temperature during the same period. Sensible heat vulnerability assessments also rely on indices of sensible heat, which refers to the heat experienced by residents. In the present study, we proposed a heat index for evaluating thermal vulnerability according to sensible heat flux, which reflects the degree of the exposure within a given environment.

Assessments of thermal vulnerability involve a series of processes [84] that comprehensively determine how sensitive an area is to the effects of heat change, and how well it is capable of adapting. In the present study, we adopted a formula to evaluate the level of thermal vulnerability. This formula applies the numerical values for the exposure, sensitivity, and adaptive capacity derived from the Analytical Hierarchy Process (AHP) based on questionnaires from disaster-related practicing professionals [81,85]; 35 of managers and employees in various fields of industries such as car, gas, electricity, and researchers in the climate change field. The questionnaire data collected from the professionals were converted using a weighing scale, ranging from 0 to 1, with the AHP [86]. The weights of three indices influencing heat vulnerability were applied to each index and the sum of these were expected to be equal to 1:

TVI = 0.398 × Exposure (E) + 0.339 × Sensitivity (S) − 0.263 × Adaptivity (A)(1)

The exposure, sensitivity, and adaptive capacity are composed of various quantitative variables. Values of each variable are standardised and added to the upper level elements (e.g., sensitivity and adaptive capacity). Each summed value is again normalised and applied to Equation (1) to calculate thermal vulnerability, the standardised value (Equation (2)) which ranges between 0 and 1. The standardisation prevented the generation of spatial deflections induced by very large or small census sites:

(2) $$\beta =\;\left[\frac{x-x_{min}}{x_{max}-\;x_{min}}\right]$$

Finally, the resulting vulnerability values are divided into five classes [82], as follows. Because there are none values on mortality rate, the range of the both sensible heat and temperature vulnerability was classified using hierarchical clustering (Appendix D) to compare with the death rate [87].

All vulnerability maps were generated based on these five classes (Table 1). Various thermal variables we used for the thermal vulnerability are described in detail in the next section. The thermal vulnerability index (TVI) [82] was determined via spatial analysis. Spatial patterns were mapped throughout the city. A set of selected variables related to thermal vulnerability was prepared and correlation analysed. The fundamental goal of utilizing an efficient set of variables was to identify both the obvious, as well as the stable, but important, relationships between the occurrence of heat events and the associated health hazard, in terms of various vulnerability variables.

These variables include population size, elderly individuals, elderly individuals living alone, people living below the poverty line, laborers, income, medical insurance budget, spatial attributes, and the number of medical institutes. Spatial attributes include vegetated areas (green), wetland and water surface (water), impervious surface, building, and road (see Appendix B Table A4). Since we researched urbanised areas at the community level, we did not include urban infrastructure, such as access to water or electrical supply and good roads, as an adaptive capacity. Although all the data were available for every year, we use the data for 2015, the latest available meteorological data collected monthly from AWS and a severity of the heat-wave. Table 2 lists the variables of vulnerability assessment from published statistical and census data. The explanations for thermal vulnerability indices are elaborated in each chapter. We analysed data for August 2015 due to the availability of certain data (e.g., health-related diseases and sensible heat) and the severity of the heatwave in 2015.

### 2.4. Sensitivity

Sensitivity is a social factor that increases vulnerability in a heat-intensive space within a city. Sensitivity was quantified based on six variables: (1) population density, (2) population of older adults over age 65 living alone, (3) population of preschool children under age 5, (4) number of heat-related illnesses, (5) population of below poverty line (BPL), and (6) population of heat-related deaths in August(Department of Welfare for Seniors, Seoul Metropolitan Government).

The number of patients with heat-related illnesses (3) corresponds to the sum of: (i) the number of patients with respiratory illnesses, (ii) the number of patients with heart disease, and (iii) the number of patients with cerebrovascular disease experiencing refractory illnesses. Data sources and descriptions are shown in Table 1. However, due to the definition of heat-wave, we also considered the second day of the heat-wave when we counted heat-related disease mortality.

The total number of items per category was weighted by each variable’s normalised value of ranking, and the proportion of elements included in the vulnerability analysis varied. Total numbers per category were divided by the total and divided by the population ratio for each administrative area to obtain a relative vulnerability assessment of the area. Demographic data were obtained from a 2015 source (Table 2).

### 2.5. Adaptive Capacity

Adaptive capacity weakens thermal vulnerability in heat-concentrated areas. Thus, there is an inverse relationship between the sum of all adaptive capacity variables, climate change exposure and sensitivity. High adaptive capacity lowers the total vulnerability score and overall spatial thermal vulnerability in areas with high concentration.

Adaptive capacity was determined based on: (1) income, (2) the number of medical institutions, and (3) the annual medical insurance budget by an administrative community (called “dong”). Income was defined as average monthly income in 2015, which was calculated by dividing household income by the population of the neighbourhood unit. We included the number of hospitals (rather than distance from the hospitals) as one factor of adaptive capacity. The reason why the number of beds was considered as an important factor is that there are hospitals at the centre of every “Dong” at the community level, which allows the residents to access the hospitals within a radius of approximately 2 km [88]. In Seoul, the number of hospitals [32] or the number of beds was more likely to affect vulnerability.

### 2.6. Exposure

In this study, the climate change exposure is composed of three variables: air temperature, sensible heat flux and landcover ratio (here, spatial attributes). Air temperature data were obtained from 38 Automatic Weather Stations (AWS) in Seoul and from 249 AWS owned by SKTech X (Seoul, Republic of Korea), a private company. Data were constructed from equipment installed at least 15 m above the ground (i.e., four stories). Sensible heat flux was calculated using the energy budget model adopted from previous studies [24]. The value was calculated using a thermodynamic model that considers the urban canyon structure. It contains landcover ratio of the study site and architectural aspects, such as the height of buildings and altitude. The ratio of five landcovers was extracted from landcover classification shape files. Area statistics for each census area were calculated using ArcGIS Desktop Release 10.2 (ESRI Inc., Redlands, CA, USA, 2014). Exposure values for each census were calculated by averaging the pixel-based estimates for each census; exposure was estimated using the standard deviation of sensible heat flux for the same census area for which atmospheric temperatures were obtained.

### 2.7. Sensible Heat Flux Estimation

Sensible heat flux was derived based on four types of heat flux [24]. We used the following energy budget model to calculate sensible heat flux based on net radiation (*Q_n_*), which was derived by Offerle [89]. All of the heat fluxes have units of W/m^2^: [89,90,91,92]:

(3) $$Q_{n}+\;Q_{F}\;=\;\;Q_{h}\;+\;\;Q_{e}\;\;+\;Q_{s}\;\;$$

The energy budget is composed of four elements, namely, the anthropogenic heat flux (Q_F_), sensible heat flux (Q_h_), latent heat flux (Q_e_), storage heat flux (Q_s_). All the models are explained in Appendix A. 

### 2.8. Thermal Disease-Related Mortality

Heat-related diseases were defined based on a previous study [63]. In this study, we included data for patients with respiratory, heat disease (ischaemic) and cerebrovascular disease to calculate thermal vulnerability. Those diseases are mostly higher than 1 (cumulative lags 0–2) of relative risk (RR) under 95% confidence intervals of mortality and extremely hot days by 66 causes of death. The number of deaths in August 2015 due to these three diseases was determined for both sexes, and each distribution was calculated and extracted among various mortality causes by using SPSS (IBM Corp. Released 2013. IBM SPSS Statistics for Windows, Version 22.0.) and R (R Core Team, 2017). In addition, we obtained raw data of heat-related diseases (Table 3) and mortality rate from Korea Centres for Disease Control and Prevention for estimating the sensitivity and comparison of the vulnerability indices.

## 3. Results and Discussion

One aim of the present study was to identify additional design criteria for improving the thermal environment based on vulnerability analysis. In our correlation analysis, we investigated the distribution and numerical value of temperature, which are existing indicators of the sensible heat vulnerability, as well as the distribution and numerical value of sensible heat flux, a novel indicator. We compared these variables in relation to the number of heat-related deaths. We also aimed to determine the spatial distribution of sensible heat vulnerability in relation to mortality rates, and whether these exhibited quantitative correspondence with the spatial distribution of temperature vulnerability in relation to mortality rate. 

### 3.1. Maps of Three Indices

We derived three kind of indices maps, sensitivity, adaptive capacity, and exposure (Figure 2). To find different variables’ impact on the indices, we use the exposure map based on sensible heat, we also created sensible vulnerability based on sensible heat flux during the day (map of Figure 3). Because the vulnerability indices are standardised values, a range of class is divided by equal interval in GIS, which is best applied to familiar data ranges, such as temperature.

The vulnerability index, which does not reflect temperature and sensible heat, shows sensitivity and adaptive capacity for both maps a) and b) (Figure 3). However, due to changes in temperature and sensible heat flux, which are included in climate change, maps of temperature or areas vulnerable to heat are different.

### 3.2. Validation Based on the Root Mean Square Error (RMSE)

We compared the distribution of heat-related deaths in August 2015 between areas with high vulnerability based on sensible heat flux and temperature. The two vulnerability distributions are shown in Figure 4. Calculations were performed based on the RMSE for each vulnerable area. We observed a correlation of 0.71 (Table 4) between vulnerable areas based on sensible heat flux and mortality rate. These findings suggest that deriving vulnerable areas based on sensible heat flux is more objective than deriving them based on temperature. Indeed, the high correlation coefficient indicates the reliability of the sensible heat flux as an index for determining thermal disease-related mortality:

(4) $$\mathrm{RMSE}:\;\sqrt{\frac{1}{n}\;\sum_{i=1}^{n}{\left(D_{i}-\;V_{i}\right)}^{2}}$$

$D_{i}$ and $V_{i}$ represent death rate and vulnerability, respectively, while *n* represents the number of administrative regions (Equation (4)). In this study, we investigated the distribution of death rates among patients with febrile illness during vulnerability assessments based on temperature and sensible heat. 

The changes in community (*Dong*) vulnerability index based on temperature and vulnerability indices based on sensible heat flux are as follows (Figure 4):

(a) The mean values of the changes in the temperature vulnerability index and the sensible heat vulnerability index were 0.25 and 0.21, respectively.
(b) The differences between the maximum and minimum values of the temperature vulnerability index and sensible heat vulnerability index were 0.43 and 0.50, respectively.
(c) The general trajectory drawn by the vulnerability index based on temperature is a kind of multi-nuclei circle. A trend line of sensible heat vulnerability index is similar to concentric circles but the trajectory of the sensible heat vulnerability index differs from the temperature vulnerability index.
(d) The trajectory of mortality rate of the community is similar to concentric circles, like the sensible heat vulnerability index.
(e) As a result, the transition trends of the community-by-*Dong* mortality rate and the sensible heat vulnerability index are similar.

We adopted this approach to validate the objectivity of these indicators. RMSE analyses provide a numerical value representing the accuracy of the model by comparing the difference between the estimated value and the actual value. For RMSE analyses, we adopted the two vulnerability values as the estimated values, whereas mortality rate was adopted as the actual value. Although RMSE analyses are associated with scale-dependent errors, the influence of such errors depends on the range of the estimated value. However, in this study, changes in sensible heat flux and temperature were standardised for the vulnerability assessment, and the mortality rate was standardised in the same way.

Our results indicated that the distribution pattern for vulnerability assessments based on a correlation coefficient value of 0.431 (0.734–0.303) for sensible heat flux is higher than that for assessments based on temperature when examining mortality rates (Table 4). In other words, sensible heat flux is more useful for estimating thermal vulnerability considering the mortality from heat-related illness than by considering temperature at the community level.

Sensible heat vulnerability index was associated with mortality rates that were quantitatively similar to those for temperature vulnerability index (Figure 5). However, the mortality rate was determined based on the average for the administrative unit of *Dong*, which may not accurately reflect the standard variables for the community.

### 3.3. Findings from Community’s Comprehensive Thermal Vulnerability Index

We obtained the indicators to improve the thermal environment based on the sensible heat flux change values of the communities associated with the thermal vulnerability index; and the mean, minimum, and maximum values were calculated, and the following results were noted:

(a) For grade 5, the average sensible heat flux level was approximately 324 w/m^2^. As a result of the good thermal environment, the first grade showed an average sensible heat flux of about 170 w/m^2^.
(b) In order to mitigate the thermal environment from grade 5 to grade 1, green space expansion requires a sustainable energy policy by mitigating the coverage rate during land cover.
(c) The following factors affect thermal vulnerability at the community level: Climate Exposure> Sensitivity> Adaptive capacity.
(d) Adaptive capacity affects negatively thermal vulnerability. Two indices, sensitivity and climate exposure, are in a positive relationship. The street views of the three highest and lowest ranked communities for sensitivity and climate exposure are shown in the Appendix D.
(e) The mortality trend reported for August 2015 appeared to reflect the sensible heat flux.

The mortality rate in August shows that communities with higher sensible heat flux had a higher thermal vulnerability (Table 5). The mortality rate was calculated considering an annual total number of mortalities in 2015 and number of August mortalities. The Oryu community had the highest number of mortalities in August; however, the annual rate was lower than those in the Wolgye and Noryiangjin communities. The annual number of mortalities in Oryu was the highest at 183.

### 3.4. Spatial Attributes and Patterns Related to Sensible Heat Vulnerability

**Figure 6:**
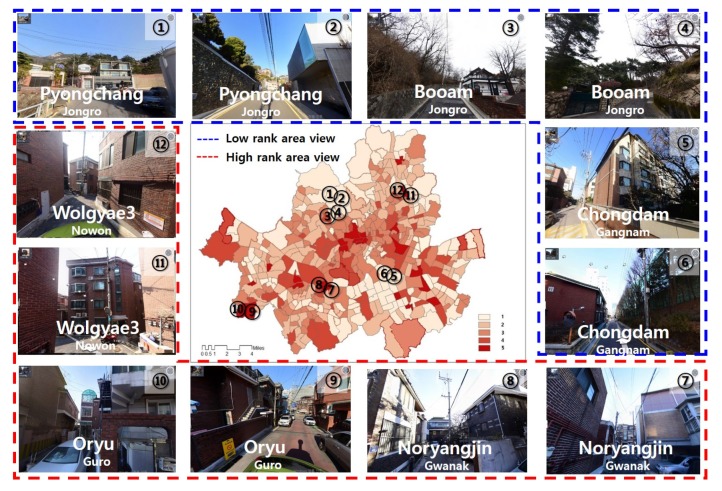
Street views of the relative highest and lowest sensible heat vulnerability. Note: Twelve street views for six communities representing the highest rank (the first through the third picture ①~⑥) and the lowest rank (436th through 438th, picture ⑦~⑫), each of which can relate its context and situation to the community’s thermal vulnerabilities.

To examine the spatial causes of sensible heat flux vulnerable communities, we observed the differences in sensible heat vulnerability between older urban areas of the city and urban residential areas (Figure 6). Areas with higher vulnerability mainly included dense residential areas and compact spatial structures. Areas with lower vulnerability mainly included low-density residential areas. The distribution of sensible heat vulnerability also differed from that for temperature vulnerability.

Spatial patterns of temperature vulnerability differed significantly among the western, northern, and southern regions of the city. The highest vulnerability values were observed in the southwest and northeast areas of the city. These areas are low-rise residential neighbourhoods with high population densities. The lowest values were observed in the northwest and southeast regions of the city, which include urban areas.

Low vulnerability values in the city’s core district may have been influenced by high building density or high income, as high-rise buildings exert a cooling effect by providing shade. In some cases, cooling is associated with high plant density and the presence of green infrastructure, parks, or other green areas [24,93]. In other cases, lower levels of impervious surfaces are associated with lower temperatures and reduced thermal vulnerability.

The spatial characteristics of communities associated with thermal vulnerability affecting the health of residents due to changes in living environment in each community are as follows (Figure 6):

(a) Seoul’s 438 “dongs” have individual placemarks based on the culture and traditions of each community. Each community expresses the thermal environment in a distinctive space, creating sensible heat mainly in the building and open space among five typical urban land cover factors. These communities (Figure 6, picture **①~⑥**) had a land cover attribute that reduced sensible heat flux. According to the previous study, when the area of green surface increased by 1%, the sensible heat flux decreased by 4.9 w/m^2^ [24]. However, an increase in impervious surface area contributed to increased sensible heat flux (Figure 6, picture **⑦~⑫**).
(b) In this study, we obtained a “street view” that had a symbolic place among the communities with relative uniqueness of thermal vulnerability. By reviewing twelve pictures as shown in Figure 6, the properties of two thermal environmental types, favourable and unfavourable areas, were reflective of the land cover types [24].

## 4. Conclusions

In the present study, we compared the distribution of sensible heat vulnerability and temperature vulnerability at the community level in order to identify an appropriate indicator that can be used to improve the thermal environment. Our findings indicated that the distribution of vulnerability as derived based on sensible heat was more similar to the distribution of mortality rate than to the distribution of vulnerability as derived based on temperature. Thus, our results demonstrate that sensible heat flux can be used as an objective indicator in the assessment of thermal vulnerability. Future studies should aim to determine how this indicator can be used to inform spatial planning and design criteria [24].

Adaptation to climate change is a holistic issue affected by social, economic, and environmental factors [94]. There are inherent limitations to using standardised values for correlation coefficients, given the differences in clinical background and personal characteristics among individuals [95]. Therefore, although the correlation between mortality and temperature was under 0.5 in the present study, one cannot conclude that this parameter is meaningless. Additional studies should aim to establish standard indicators for improving the thermal environment at the community level. 

Our results suggest that difficulties in reflecting the spatial characteristics that influence thermal vulnerability can be addressed using measures of heat flux. The sensible heat vulnerability index can, therefore, be useful to developing practical methods for improving the thermal environment and estimating another thermal barometer, sensible heat flux in community scale. The proposed variable, sensible heat flux, could be further employed in examining the thermal vulnerability in the community level. Further, a suggested technique on vulnerability to heat at spatial resolutions finer than the regional scale is useful to assist decision makers with mitigation of the vulnerability in the preparation for and response to extreme heat events. 

And it helps representing spatial characteristics of a community, where heat vulnerable areas are derived, based on sensible heat flux. This research contributes to considering the way to create an implemental tool, a sensible heat vulnerability index mapping method, and designing standards for thermal environment improvement. Therefore, we encourage the spatial fine resolution and an approach with the new variable to assess the vulnerability. We also suggest that this methodology has an indirect effect on spatial sustainability.

## Figures and Tables

**Table 1:** Level of thermal vulnerability index (TVI).

Level	Criteria	Range
1	Seriously vulnerable to heat	0.00–0.08
2	Vulnerable to heat	0.08–0.32
3	Mild	0.32–0.49
4	Not vulnerable to heat	0.49–0.76
5	Seriously not vulnerable to heat	0.76–1.00

**Table 2:** Thermal vulnerability index (TVI).

Index	Variable	Data Description	Year	Data Source
Sensitivity	Population density	Inhabitants per area *	2015	Seoul open dataset **
	Older adults (over 65) living alone	Inhabitants per area * above 65 years old	2015	Seoul open dataset **, Dept. of welfare for seniors, Seoul
	Population of under 5	Inhabitants per area * under 5 years old	2015	Seoul open dataset **
	Heat-related illness	Inhabitants per area * with heat-related illness	2015	Seoul open dataset **
	Population below poverty line (BPL)	National Basic Livelihood Act recipients per area *	2015	Seoul open dataset **
	Heat-related death	Inhabitants per area * with heat-related death	2015	Seoul open dataset **
Adaptive capacity	Hospitals	Number of medical institutes	2015	Seoul open dataset **
	Income	Monthly income	2015	KOSIS ****
	Medical insurance budget	Annual budget	2015	Seoul open dataset **
Exposure	Daytime air temperature	Average daytime *** temperature	2015	SKTech X (249 stations)
	Daytime sensible heat flux	Average daytime *** sensible heat flux	2015	Estimation
	Spatial attributes (see Figure A1)	Subdivided land cover classification map (green, wetland, impervious surface), building shp. File, widths of roads shp. File	2015	Ministry of Environment, Statistical Geographic Information Service, Seoul Information Communication Plaza

* area: area of Dong, ** Seoul open dataset: http://data.seoul.go.kr/, *** Daytime: 12:00~16:00, **** KOSIS: Korean Statistical Information Service.

**Table 3:** Heat-related diseases (Unit: ratio).

Heat-Related Disease		*RR*
Respiratory	Pneumonia	1.2
	Chronic lower resp. dis.	1.2
	Other resp. dis.	1.3
Cardiovascular	Heart: Ischaemic	1.2
	Cerebrovascular	1.2
	Atherosclerosis	1.4
	Hypertensive	1.3
Digestive system	Ulcers	1.0
	Liver diseases	1.2
RR: Basagaña et al. [63]		

**Table 4:** Correlation of temperature and Q_h_ vulnerability &RMSE of temperature, Q_h_ vulnerability and mortality.

	Correlation	RMSE **	Average Error
Max.* Temperature (˚C)	0.303	0.229241081	−0.20112
Max.* Sensible Heat flux (W/m^2^)	0.734	0.184579627	−0.17102

Max *: maximum; RMSE **: Root mean square error for mortality rate.

**Table 5:** Top three-rank and bottom three-rank to sensible heat vulnerability index.

Community	SHVI * (Rank)	Sensible Heat Flux(W/m^2^)			Sensitivity	Adaptive Capacity	Exposure	Mortality ** (Total (n)/Mortality Rate (ratio)	Attributes
		Mean	Max	Min					
Wolgea 3	1	207.60	511.50	133.27	0.93	0.13	0.79	9 (0.08)	Old town
Oryu	2	200.30	536.79	105.57	0.91	0.27	0.87	12 (0.07)	mixed residential district
Noryangjin	3	241.98	489.70	116.91	0.82	0.07	0.72	10 (0.08)	Farmers & fishery market
Cheongdam	436	216.23	292.09	104.58	0.17	1.0	0.11	1 (0.01)	New developed residential area
Booam	437	197.47	305.49	127.87	0.17	0.67	0.15	1 (0.02)	Old low-rise residential area
Pyungchang	438	180.79	304.79	122.99	0.28	0.84	0.15	1 (0.01)	Old low-rise residential area

SHVI *: Sensible heat vulnerability index; Mortality **: rate of mortality in August—a ratio to amount of an annual mortality (ratio).

**Figure A1:**
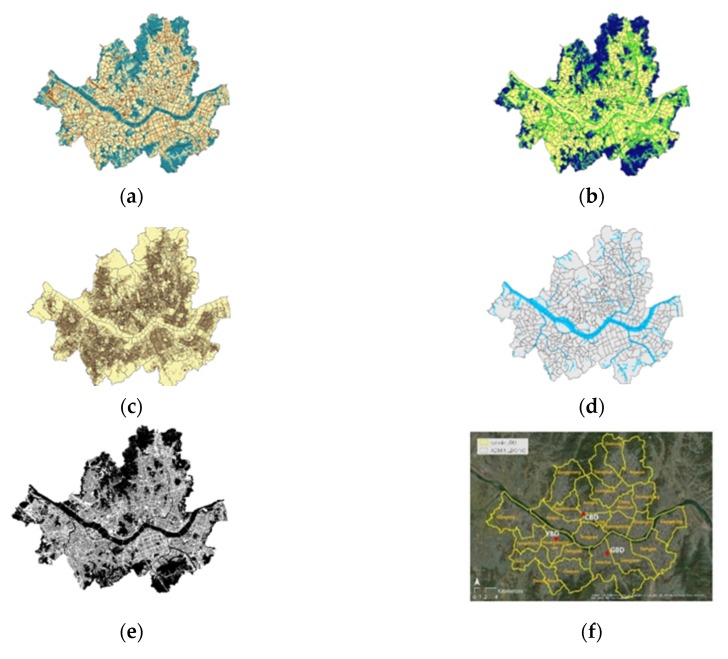
Input data: (**a**) impervious surfaces; (**b**) green spaces; (**c**) buildings; (**d**) water and wetlands; (**e**) roads; and (**f**) administrative map.

**Figure A3:**
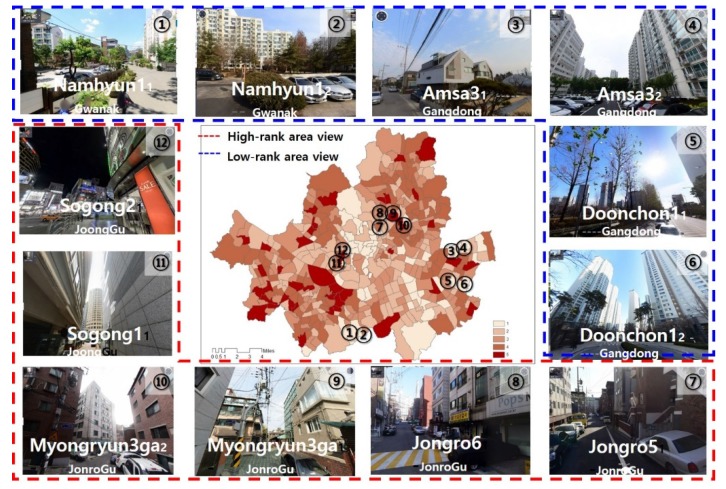
Street views of top three-ranked and bottom three-ranked communities for thermal sensitivity.

**Figure A4:**
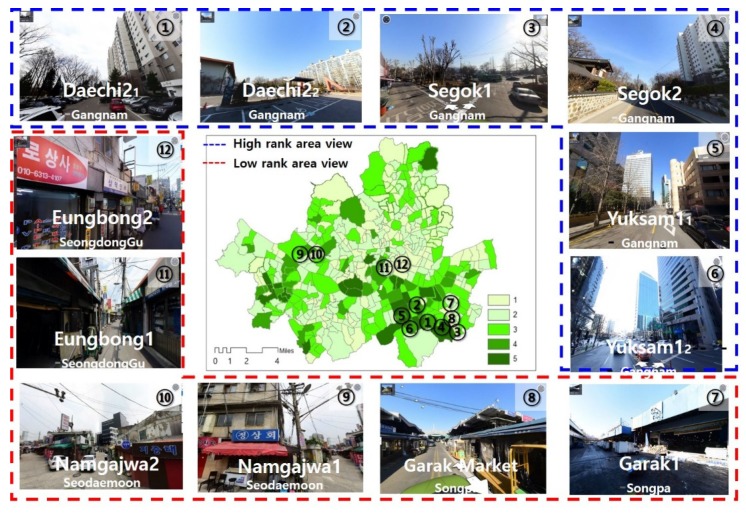
Street views of top three-ranked and bottom three-ranked communities for thermal adaptive capacity.

**Figure A5:**
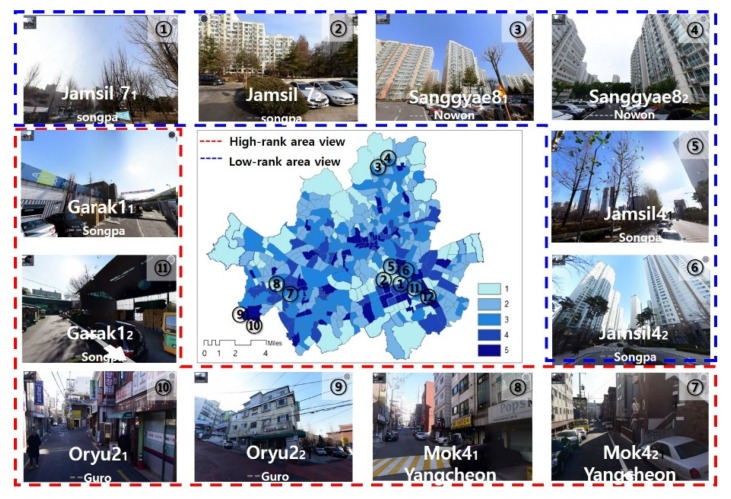
Street views of top three-ranked and bottom three-ranked communities for climate exposure.

## Appendix A. Sensible Heat Flux Estimation

Here, QF, Qh, Qe, Qs, and ΔQA refer to the anthropogenic heat flux, sensible heat flux, latent heat flux, storage heat flux, and horizontal heat flux, respectively. The net radiation was derived from the urban energy balance in (1), as described by Offerle et al. [89]. All heat fluxes are presented in units of W/m^2^:

(A1) $$\Delta Q_{S}=\sum_{i=1}^{l}f_{i}\times (a_{1i}Q_{n}+a_{2i}\Delta Q_{n}+a_{3i})$$

$f_{i}$: land cover ratio (unit: ratio)

i: green cover, water cover, impervious land, building cover, and road cover

$\frac{\gamma }{s}=$−0.00003178 $\times temp^{3}+0.03\times temp^{2}-0.092\times temp^{}+1.463\;$.

The storage heat flux in (2) was derived from an equation considering the land cover ratio and empirical coefficients. Here, γ is a psychrometric constant, and s is the slope of the curve of saturation vapour pressure versus temperature:

(A2) $$Q_{h}=\left[\frac{\left\{\left(1-\propto \right)+\left(\frac{\gamma }{s}\right)\right\}}{\left\{1+\left(\frac{\gamma }{s}\right)\right\}}\;\right]\;\times \;(Q_{n}-\;\;\Delta \;Q_{S})\;+20$$

(A3) $$Q_{e}=\left[\frac{\propto }{\left\{1+\left(\frac{\gamma }{s}\right)\right\}}\;\right]\;\times \;(Q_{n}-\Delta Q_{S})+20$$

α :an empircal parameter related to the moisture status of the surface.

Both sensible heat flux (3) and latent heat flux (4) were estimated using the model developed by Holtsalg and Van Ulden [90]. In these equations, 20 (W/m^2^) is an empirical constant. Both α and 20 (W/m^2^) were determined based on the Penman Monteith approach [96]:

(A4) $$Q_{F}\;=\;6.8\;(T_{C}-T_{d})\;+\;12\;(\mathrm{for}\;T_{d}\;\leq \;T_{C}),$$

where *T_C_* represents the maximum daily temperature (unit: K) and *T_d_* represents the mean daily temperature (unit: K).

The anthropogenic heat flux (5) was estimated using a model that considers temperature. Advection (Δ*Q_A_*) was negligible at the six investigated sites on the day of measurement because we chose a day with a wind speed of approximately 0 m/s.

The land cover ratio ($f_{i}$) was calculated using the relative area occupied by each type of land cover within a 100 m ×100 m grid (Table A1 and Table A2.). Empirical land cover coefficients and anthropogenic heat flux at the neighbourhood scale were calculated as previously described [24].

**Table A1:** Empirical land cover coefficients.

Land Cover Coefficient	*a*_1_ (ratio)	*a*_2_ (h)	*a*_3_ (W/m^2^)
Green	0.34	0.31	−31
Building	0.07	0.06	−5
Impervious	0.83	0.4	−54.2
Water	0.5	0.21	−39.1
Road	0.61	0.41	−27.7

Source: Grimmond and Oke [97], Roberts and Oke [98].

**Table A2:** Anthropogenic heat flux at the neighbourhood level.

Neighbourhood	LCZ *	Anthropogenic Heat Flux (W/m^2^)
High density, city centre	1, 2	100–1600 **
Medium density, city centre	3	30–100 **
Low density, open, low-rise	6	5–50 **
Open, high-rise	4	26–80 ***
Green (low-planted), Water	D, G	-

LCZ: * Local Climate Zone, Source: ** Oke et al. [99]; *** Pigeon et al. [91].

## Appendix B. Data

The hourly heat flux was calculated using the data of air temperature, atmospheric pressure, and relative humidity collected from 287 stations in total, including 38 meteorological stations in Seoul and 249 SKTech X meteorological stations. We typified the heat flux and land cover data (Figure A1, Table A3) on a day with clear weather, low cloud cover, and peak air temperature in August (Table A3) [24]. We also created a 100 m × 100 m grid to map the thermal environment and thermal distribution data.

**Table A3:** Metrology and spatial attributes (land cover factors): data and sources.

Classification	Input Data	Source
Meteorological data for heat flux distribution	-Air temperature, relative humidity, cloud cover, saturated water vapour pressure	-Korea Meteorological Administration (38 stations) -SKTech X (249 stations)
Spatial attributes	-Subdivided land cover map (green spaces, wetlands, impervious surfaces) -shp file of Seoul administrative district – building .shp file -shp file depicting the widths of roads -shp file depicting the width of roads	-Ministry of Environment, -Statistical Geographic Information Service (SGIS), -Seoul Information Communication Plaza

**Table A4:** Correlation matrix for TVI variables.

	*A*	*B*	*C*	*D*	*E*	*F*	*G*	*H*	*I*	*J*	*K*	*L*	*M*	*N*	*O*	*P*	*Q*	*R*	*S*	*T*	*U*	*V*	*W*
A	1.00																						
B	0.15	1.00																					
C	0.23	0.09	1.00																				
D	0.19	0.57	0.07	1.00																			
E	0.20	0.57	0.07	0.99	1.00																		
F	0.07	0.18	0.71	0.06	0.06	1.00																	
G	0.16	0.78	0.01	0.69	0.69	0.14	1.00																
H	0.01	0.19	0.03	0.20	0.21	0.02	0.27	1.00															
I	0.13	0.35	0.01	0.55	0.55	0.14	0.70	0.11	1.00														
J	0.08	0.02	0.03	0.07	0.07	0.00	0.04	0.06	0.11	1.00													
K	0.07	0.07	0.01	0.14	0.13	0.02	0.27	0.05	0.42	0.01	1.00												
L	0.19	0.11	0.12	0.11	0.10	0.08	0.04	−0.02	0.17	0.07	0.10	1.00											
M	0.04	0.04	0.03	0.19	0.19	0.07	0.00	0.04	0.07	0.12	0.24	0.04	1.00										
N	0.38	0.05	0.03	0.03	0.03	0.03	0.04	0.04	0.20	0.12	0.13	0.42	0.00	1.00									
O	0.43	0.11	0.11	0.09	0.08	0.10	0.02	0.02	0.28	0.15	0.19	0.73	−0.04	0.66	1.00								
P	0.10	0.35	0.06	0.35	0.36	0.29	0.42	0.12	0.29	0.00	0.05	0.06	0.33	−0.02	0.05	1.00							
Q	0.11	0.19	0.04	0.31	0.32	0.01	0.16	−0.04	0.02	0.05	0.50	0.01	0.63	−0.02	0.03	0.15	1.00						
R	0.58	0.04	0.03	0.09	0.09	0.05	0.08	0.06	0.14	0.16	0.05	0.24	−0.03	−0.48	0.70	0.02	0.04	1.00					
S	0.50	0.12	0.05	−0.03	0.03	0.05	0.00	0.04	0.25	0.15	0.17	0.73	−0.03	0.64	0.95	0.06	0.04	0.72	1.00				
T	0.30	0.12	0.19	0.04	0.04	0.06	0.06	−0.02	0.09	0.08	0.10	0.64	0.03	−0.04	0.19	0.06	0.05	0.39	0.15	1.00			
U	0.26	0.15	0.04	−0.10	0.10	0.08	0.08	−0.10	0.07	0.15	0.02	0.23	0.03	−0.40	0.35	0.01	0.00	0.02	0.38	0.06	1.00		
V	0.33	0.02	0.08	−0.15	0.15	0.09	0.07	−0.08	0.13	0.14	0.01	0.49	0.08	0.46	0.69	0.08	0.13	0.73	0.65	0.16	0.01	1.00	
W	0.62	0.45	0.25	0.35	0.35	0.36	0.41	0.06	0.19	0.33	0.06	0.35	−0.25	0.46	0.67	0.17	0.28	0.68	0.69	0.17	0.32	0.54	1

Note: A ~ W—Population (pop.) density, pop. of over 65 living alone, pop. of under 5, pop. of heat-related illness, pop. of below poverty line, pop. of mortality in August, *pop. of over 65, pop. of single household, pop of under Highschool*, distance to hospitals, *number of hospitals*, income, amount of medical insurance, temperature, sensible heat flux, *natural disaster, wind speed in August*, green, building, impervious surface, water, road, vulnerability (Variables in italic font are excepted for the Table 2).

## Appendix C. Hierarchical Clustering Analysis

The level range of vulnerability standardised value is divided by hierarchical clustering. According to the hierarchical clustering analysis, each level is ranged as; 1 (0~0.08), 2 (0.08~0.32), 3 (0.32~0.49), 4 (0.49~0.76), 5 (0.76~1.00).
